# The effects of ambient particulate matter air pollution on platelets and hemostasis

**DOI:** 10.3389/fpubh.2024.1410406

**Published:** 2024-07-18

**Authors:** Sasinee Hantrakool, Maitree Sriwichai, Banphot Shaengkhamnang, Nipapan Leetrakool, Piangrawee Niprapan, Sawaeng Kawichai, Sitapak Wannakul, Noppamas Panyasit, Pakinee Tuntivate, Ornkamon Wongtagan, Rungrote Natesirinilkul, Pimpisid Koonyosying, Phichayut Phinyo, Teerachat Punnachet, Nonthakorn Hantrakun, Pokpong Piriyakhuntorn, Thanawat Rattanathammethee, Chatree Chai-Adisaksopha, Ekarat Rattarittamrong, Adisak Tantiworawit, Lalita Norasetthada, Somdet Srichairatanakool

**Affiliations:** ^1^Division of Hematology, Department of Internal Medicine, Faculty of Medicine, Chiang Mai University, Chiang Mai, Thailand; ^2^Blood Bank Section, Faculty of Medicine, Chiang Mai University, Chiang Mai, Thailand; ^3^Research Institute for Health Sciences, Chiang Mai University, Chiang Mai, Thailand; ^4^Division of Hematology/Oncology, Department of Pediatrics, Faculty of Medicine, Chiang Mai University, Chiang Mai, Thailand; ^5^Department of Biochemistry, Faculty of Medicine, Chiang Mai University, Chiang Mai, Thailand; ^6^Center for Clinical Epidemiology and Clinical Statistics, Faculty of Medicine, Chiang Mai University, Chiang Mai, Thailand

**Keywords:** particulate matter, coagulation, hemostasis, von Willebrand factor, platelet function

## Abstract

**Introduction:**

Elevated ambient pollution exposure is potentially linked to thromboembolism. However, the mechanisms by which particulate matter (PM) interferes with the balance of hemostatic system remain unclear. This study investigates PM-mediated hemostatic changes in individuals across unique seasonal variations of ambient pollution.

**Methods:**

This prospective study was conducted between February and July 2020 during alterations in ambient pollution in Chiang Mai, Thailand. Blood tests from 30 healthy subjects were assessed at four-week intervals, four times in total. Various coagulation tests, including prothrombin time (PT), activated partial thromboplastin time (aPTT), von Willebrand factor (vWF), platelet count, and platelet functions, were evaluated. A mixed-effects model was used to analyze the impact of high PM2.5 and PM10 on hemostatic parameters.

**Results:**

Thirty male subjects with mean age of 38.9 ± 8.2 years, were included. High levels of PM2.5 and PM10 were significantly associated with PT shortening, with no such effect observed in aPTT. PM2.5 and PM10 values also positively correlated with vWF function, while vWF antigen levels remained unchanged. Soluble P-selectin showed a strong positive association with PM2.5 and PM10 levels. Platelet function analysis revealed no correlation with PM values.

**Conclusion:**

Short-term exposure to elevated PM2.5 and PM10 concentrations was linked to shortened PT and enhanced vWF function in healthy individuals. Exploring the impact of these changes on clinically relevant thrombosis is crucial. Additional studies on the pathogenesis of pollution-related thrombosis are warranted for maintaining good health.

## Graphical abstract

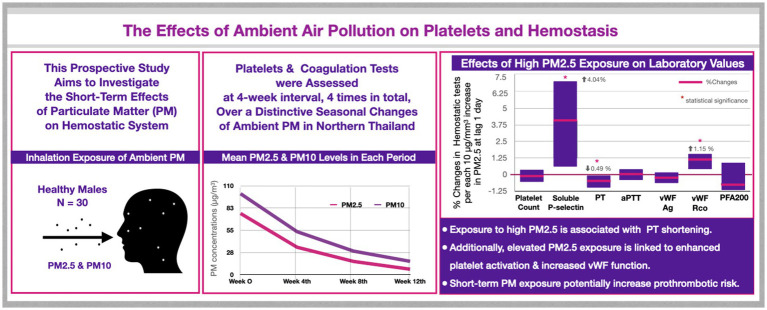

## 1 Introduction

Increasing ambient particulate matter (PM) emerges as a significant global health concern (1, 2). Numerous data sets substantiate that the inhalation of fine PM enhances various inflammatory cytokine activations, induces reactive oxygen species resulting in tissue damage, and may contribute to thrombosis (3–11). Further investigation reveals that both short-term and long-term exposure to elevated levels of ambient PM is associated with an increased incidence of adverse clinical outcomes, including myocardial infarction (12–15), cerebrovascular accidents (15–17), thromboembolism (18–20), and cancer (21–24). It is imperative to acknowledge that the relationship between PM exposure and these health outcomes is intricate and multifaceted. Moreover, exposure to elevated ambient PM could markedly increase the risk of mortality, emerging as a leading cause of death globally (1, 2).

Chiang Mai is a major city in northern Thailand where residents experience a distinctive seasonal pollution phenomenon that repeats annually. The initial trimester witnesses rising temperature and aridity, potentially leading to forest fires. Concurrently, the practice of agricultural burning contributes to ambient air pollution. Furthermore, the topography of the region, characterized by a basin surrounded by mountains, amplifies the prevalence of haze and smoke. The onset of smoke is typically observed in February, intensifying through March, gradually subsiding in April and May, and ultimately dissipating with the arrival of rainy season in June and July due to meteorological changes (25–28). This unique trait of seasonal pollution recurs on a yearly basis (29–31). Much evidence supports an increased incidence of respiratory problems, lung cancer, cardiovascular diseases, and thromboembolism associated with the seasonal smoke in this region (20, 32–38).

Outdoor air pollution consists of polycyclic aromatic hydrocarbons, sulfate, nitrate, organic compounds, metals, and various toxic substances. This pollution results from forest fires, agriculture burning, traffic vehicles, and industries (39). The past evidence indicates that inhalation of PM with a diameter less than 2.5 μm (PM_2.5_), and 10 μm (PM_10_) triggers pulmonary and systemic inflammation, oxidative stress, and vascular injury, leading to tissue factor and platelet activation and ultimately resulting in a hypercoagulable state (40, 41). Previous reports demonstrate that exposure to an elevated level of fine PM could induce inflammation and result in atherosclerosis (42–44). It has been reported that each 10 μg/m^3^ increase in PM_2.5_ correlates with a 16% increased mortality from acute coronary syndrome and a 14% increased mortality from stroke (45). Various studies also reveal adverse cardiovascular outcomes and venous thromboembolism related to elevated PM (12, 14, 15, 41, 46–50).

To date, several unanswered questions persist regarding the impact of elevated PM on the prothrombotic state in healthy individuals and populations at risk. Are there differences in outcomes between acute, subacute, and chronic impacts of PM exposure? Does it have a cumulative or delayed effect on the prothrombotic state? Among various noxious ambient PM, which specific organic or inorganic compounds have the most unfavorable impact? To address these uncertainties, conducting experimental studies in human seems unethical and impossible. Consequently, numerous animal studies were conducted in distinctive ways, including using PM from different sources, concentrations, routes of exposure, and evaluating short-term and long-term cumulative effects. Previous data from *in vitro* and *in vivo* studies showed that PM can induce platelet aggregation, enhance von Willebrand factor (vWF), shorten coagulation tests such as prothrombin time (PT), activated partial thromboplastin time (aPTT), and thrombotic occlusion time (3, 5, 51–57). In addition, increased plasminogen activator inhibitor-1 (PAI-1), which inhibits fibrinolytic activity, is associated with high ambient PM, this may lead to significant thrombosis (11, 55, 56). However, a limited number of clinical studies have focused on hemostasis affected by pollution. Information on how air pollution is involved in a prothrombotic state remains unclear.

We investigate the alteration of laboratory parameters, including the biomarkers of platelet activation (soluble P-selectin), platelet function, vWF, PT, and aPTT in healthy individuals exposed to different levels of ambient PM through the unique meteorological changes in Chiang Mai, Thailand. This exploration aims to discern the short-term effects of ambient PM on human hemostasis.

## 2 Materials and methods

### 2.1 Study participants

This prospective study was conducted at Chiang Mai University, Thailand, and received approval from the Institutional Research Ethics Committee (MED-2562-06732). We recruited 30 platelet apheresis donors at the Blood Bank Center, Maharaj Nakorn Chiang Mai Hospital, located in the central city of Chiang Mai, Thailand from February to July 2020. Eligible participants were Thai males aged 18–50 years, residing, and conducting daily routines within the central city of Chiang Mai. The distance between their residences and the Blood Bank Center was less than 20 km. They were in good health, not taking any medication, and met all criteria for platelet apheresis donors. After obtaining informed consent, we recorded basic information, including age, body weight, height, address, and blood groups. Blood samples were collected during platelet donation at four-week intervals, four times in total, spanning the seasonal changes of ambient pollution. This was done to assess alterations in hematologic parameters and coagulation tests. Exclusion criteria included participants who had been outside Chiang Mai city within 1 week before each study visit. All participants were encouraged to use protective measures, such as face masks and air purifiers, as part of their daily routines. The study took place at the beginning of the COVID-19 pandemic, resulting in some participants being unable to complete all study visits due to lockdowns, which led to missing data. To minimize potential confounding effects on hemostatic parameters, individuals who tested positive for SARS-CoV-2 were excluded from the study. This study was conducted before COVID-19 vaccines were available in Thailand; therefore, none of the participants were vaccinated during the study period.

### 2.2 Blood collection and laboratory analysis

Peripheral venous blood samples were obtained using 16-gauge needles by proficient medical technologists. Blood samples were collected in three types of vacuum tubes containing 3.2% sodium citrate, EDTA, and clotted blood for various analyses. After gentle mixing, the collection tubes were promptly dispatched for assessing the complete blood cell count and various coagulation tests, encompassing the PT, aPTT, platelet function analysis, soluble P-selectin, von Willebrand factor antigen (vWF:Ag), and ristocetin cofactor assay (vWF:Rco).

Citrated blood samples were submitted for PT and aPTT analysis. Platelet function was assessed using the INNOVANCE^®^ PFA-200 System with Collagen/Epinephrine and Collagen/ADP test cartridges (Siemens Healthineers, Erlangen, Germany) within 2 h of blood sampling to ensure the accuracy of the measurements. Clotted blood was promptly separated into red blood cells and serum through centrifugation at 3,000 rpm for 10 min within 45 min post-collection. Sample fractions were aliquoted and stored at −80°C prior to analysis. vWF:Ag analysis was performed in plasma utilizing an immunoturbidimetric assay (ACL TOP500, IL Coagulation Analyzer). vWF:Rco determination was performed using the aggregation method on an automated coagulation analyzer (CS-2500, SYSMEX). Soluble P-selectin determination was conducted through enzyme-linked immunosorbent assays (Cloud-Clone Corp., Wuhan, Wuhan, China).

### 2.3 Air pollution measurements

Air pollution data were provided by the Research Institute for Health Sciences, Chiang Mai University (RIHES), in collaboration with the Pollution Control Department (PCD), Ministry of Natural Resources and Environment, Thailand. These organizations established several monitoring stations to cover most areas in the central city, suburbs of Chiang Mai and the northern part of Thailand. The cooperation also suggest precautionary warnings for residents in these areas to be more vigilant and protect themselves from severe air quality deterioration.

Data included hourly concentrations of PM_2.5_ and PM_10_, CO, NO_2_, and O_3_ using the small sensor photodiode detector (PMS7003, Beijing Plantower Co., Ltd., Beijing, China) that utilizes the light scattering technique. The data were adjusted for relative humidity before reporting. The daily meteorological data, including air temperature, humidity, and wind velocity, were obtained from the Northern Meteorological Center, Chiang Mai, Thailand.

We calculated the mean daily ambient PM recorded at the Central Air Quality Monitoring Station at Sri Phum, the central station located in the city center of Chiang Mai, Thailand. It is located 1 km away from the Blood Bank Center. Additionally, PM concentrations at the nearest air monitoring station of each participant’s residence were observed and showed a strong correlation with the values obtained from the central station in the city center (*r* > 0.9).

There is frequently a time delay between exposure to ambient air pollution and the manifestation of adverse health outcomes. Furthermore, assessing cumulative exposure over a specified period can result in diverse health effects. Consequently, evaluating lag days helps capture these cumulative effects and contributes to a more comprehensive understanding of the association between air pollution and health outcomes. Thus, we calculated the mean concentrations of ambient air pollution over the lag period of one to 7 days from the date of blood collection to assess the delay and short-term cumulative effects of certain pollutions on coagulation tests. The term “mean PM_2.5_ level in the 5 lag days” indicates the average PM_2.5_ level monitored over a preceding five-day period, and we incorporated this value into our data analysis.

### 2.4 Statistical analysis

Basic characteristics of the participants were reported as mean ± SD, median, and interquartile range. Mean values of daily PM_2.5_, PM_10_ measured at the Central Air Quality Monitoring Station were analyzed, compared to PM reported from the nearest air monitoring station of the participant’s residences using the Pearson correlation test. The analysis revealed a strong correlation between them (*r* > 0.9). We selected the mean PM_2.5_ and PM_10_ at lag one to 7 days prior to the study visits of each individual and incorporated these values into the analysis to explore the short-term cumulative effects of PM on laboratory outcomes. Mean PM_2.5_ and PM_10_ along with other meteorological data at lag 1 day of each study visit at 0, 4, 8, 12 weeks, were revealed and presented in a descriptive manner.

The values of platelet count and hemostatic biomarkers of the participants at the beginning of the study were defined as the baseline. The laboratory parameters measured at 4, 8, and 12 weeks following the baseline were compared to the baseline using the Student’s *t*-test.

Linear mixed-effects models with a random intercept were used to evaluate the change in hemostatic parameters compared to the cumulative average of air pollution with a lag of one to 7 days, aiming to assess the short-term delay and cumulative effects of PM on hemostasis. We controlled for potential confounders, such as age, body weight, height, and blood group, in our linear mixed-effects models to minimize bias and provide more accurate estimates of the effects of PM exposure on hemostatic parameters. We report the mean percentage change, along with a 95% confidence interval, in the hemostatic parameters associated with each 10 μg/mm^3^ increase in the concentration of PM. Statistical analyses were performed using Stata 17 (StataCorp LLC, United States).

## 3 Results

Among 30 subjects, 26 completed the full study with four visits, two subjects had three visits, and the remaining two subjects had only two visits. The mean age was 38.9 ± 8.2 years. The basic characteristics of the participants are summarized in Table 1.

**Table 1:** Basic characteristics of the participants.

Characteristics	Participants (*N* = 30)	
Age, years (mean, SD)	38.9	8.2
Weight, kg (median, (IQR1, IQR3))	73	(68, 85)
Height, cm (median, (IQR1, IQR3))	173	(168, 175)
Blood group (N, %)		
A	6	20.0
B	10	33.3
O	13	43.3
AB	1	3.3
Distance between each participant’s home and the Central Air Monitoring Center, km (mean, range)	3.64	0.1–11

**Figure 1:**
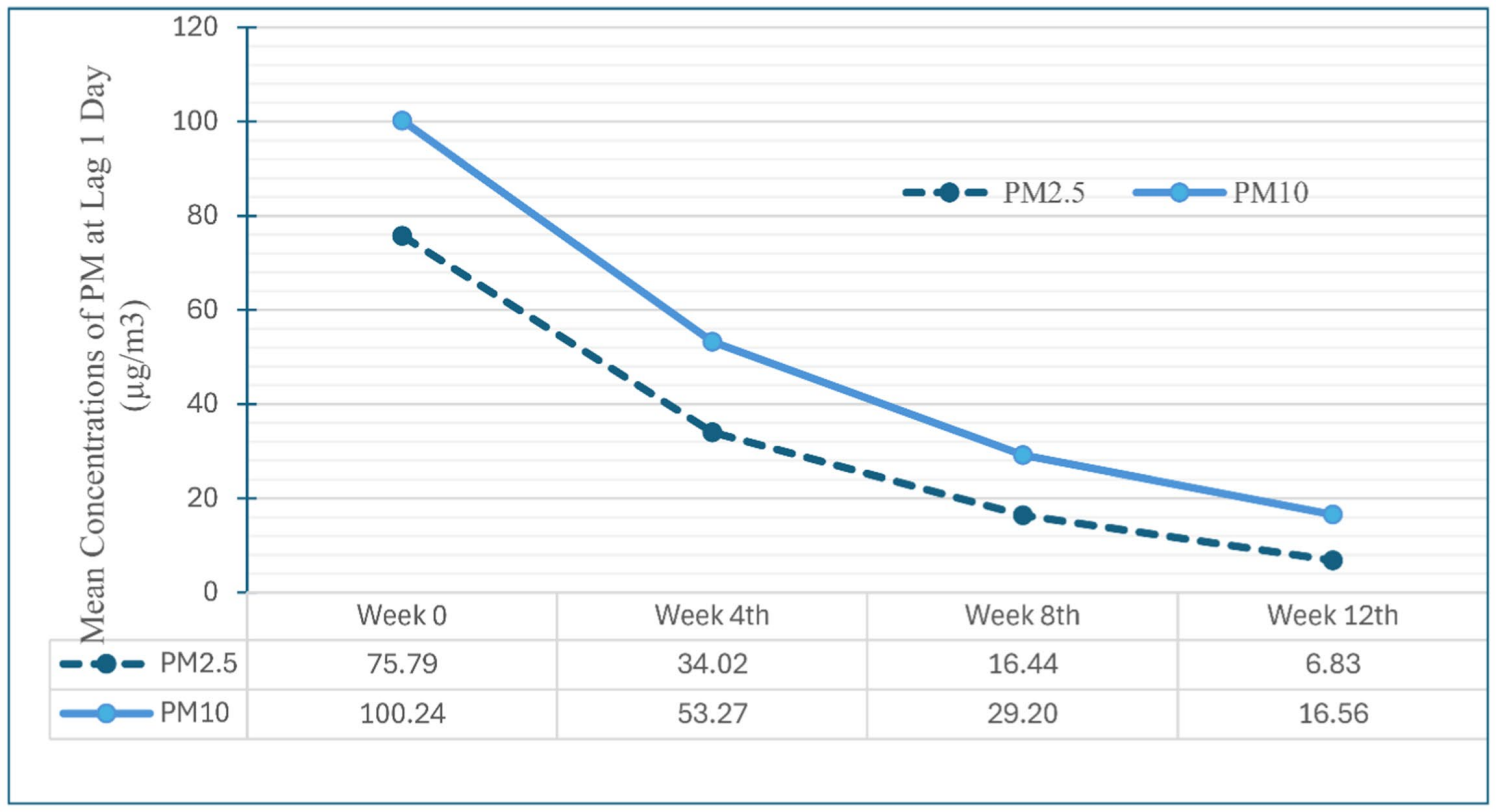
Mean concentrations of ambient PM_2.5_ and PM_10_ at lag 1 day in each visit.

The mean distance from the participants’ homes to the Central Air Quality Monitoring Station was 3.64 km, range 0.1–11 km. We observed a decline in the mean concentration of PM_2.5_ and PM_10_ over the course of the study, as shown in Figure 1. PM_2.5_ and PM_10_ levels exhibited a strong correlation (*r* > 0.9). The concentrations of ambient air pollution for each participant in each study visit are summarized in Supplementary Table S1.

**Figure 2:**
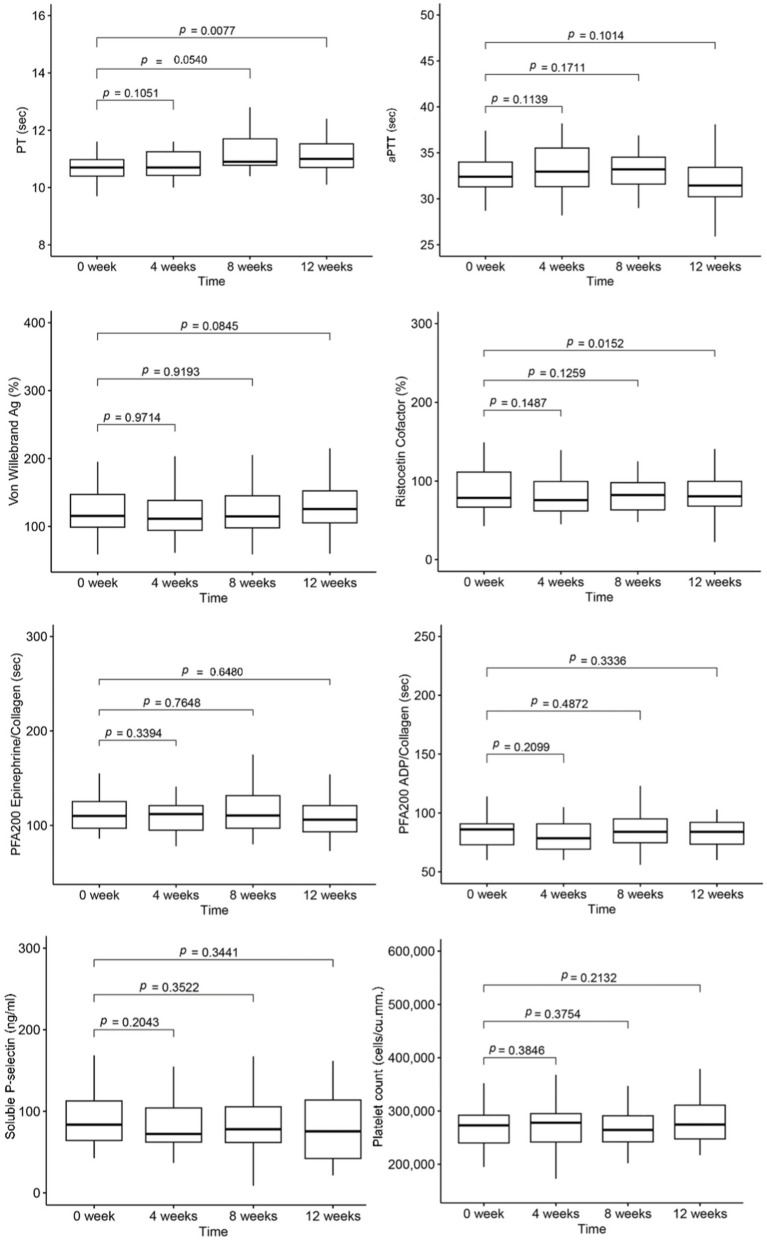
Mean values of platelet count and hemostatic parameters at each study period.

The mean values of platelet count and hemostatic tests for each visit are presented in Figure 2. Over the 12-week study period, despite falling within the normal range, the mean PT value at the 12^th^ week was slightly longer than the baseline value. However, the mean levels of aPTT showed no statistical difference. Additionally, there was a decrease in von Willebrand function, as determined by the ristocetin cofactor assay at the 12th week, while the levels of vWF antigen remained unchanged. The details of blood cell count and hemostatic parameters for each study period are provided in Supplementary Table S2.

We employed a mixed-effects model to analyze the influence of ambient air pollution on blood cell count and hemostatic biomarkers. We assessed both delayed and cumulative effects of PM over the preceding 1 to 7 days. No association was found between Hb or platelet count and PM levels.

In terms of hemostatic parameters, PT exhibited a significant negative correlation with PM_2.5_ and PM_10_ at a lag of five to 7 days. Specifically, PT values decreased by 0.62% [95% CI: 0.021, 1.22], 0.67% [95% CI: 0.06, 1.29], and 0.69% [95% CI: 0.06, 1.31] for each 10 μg/m^3^ increase in PM_2.5_ at the lag of five to 7 days, respectively. Similarly, PT values decreased by 0.51% [95% CI: 0.01, 1.01], 0.54% [95% CI: 0.04, 1.05], and 0.55% [95% CI: 0.040, 1.06] for each 10 μg/m^3^ increase in PM_10_ at the lag of five to 7 days. However, aPTT levels did not show any association with the values of PM_2.5_ and PM_10_.

The levels of vWF antigen did not show a correlation with PM but exhibited a negative association trend. Conversely, vWF function demonstrated a positive correlation with the levels of both PM_2.5_ and PM_10_ at the lag of one to 7 days.

**Figure 3:**
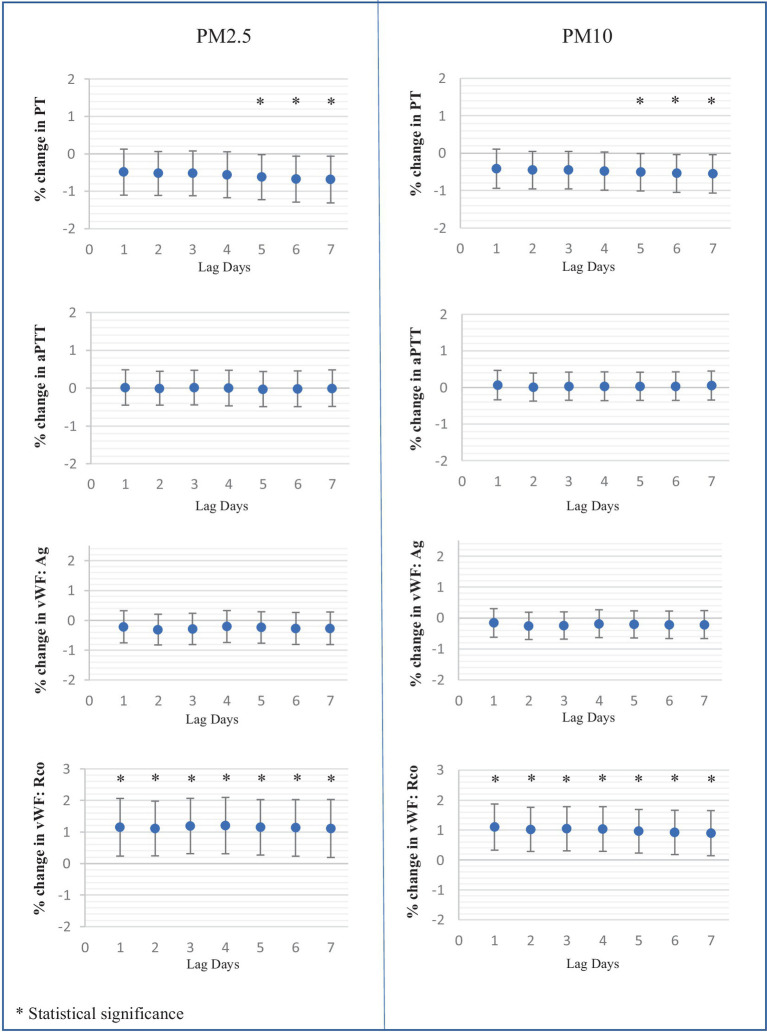

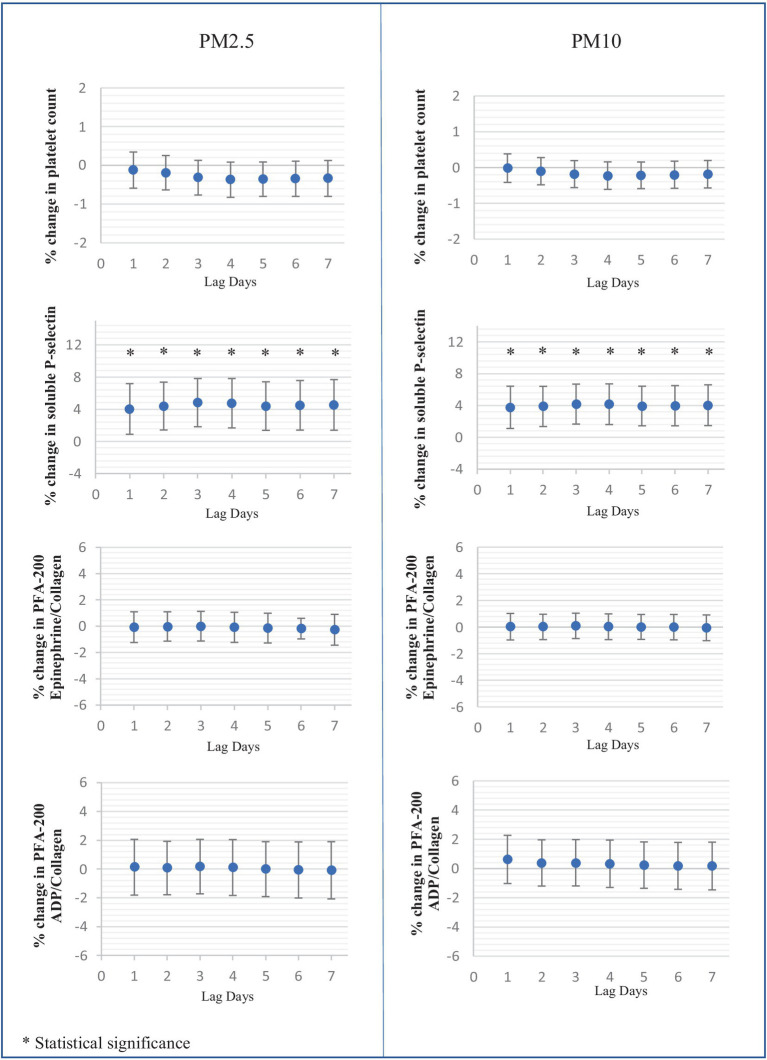
Percent changes in platelet and hemostatic parameters associated with each 10 μg/mm^3^ increase of average ambient air pollution including PM_2.5_, PM_10_ at lag days 1 to 7.

Soluble P-selectin exhibited a marked association with both PM_2.5_ and PM_10_ levels. Over the lag of one to 7 days, soluble P-selectin levels increased by 4.03 to 4.81% for each 10 μg/m^3^ rise in PM_2.5_, and by 3.78 to 4.18% for each 10 μg/m^3^ increase in PM_10_. However, the platelet function analyzer-200 system (PFA-200) using Collagen/Epinephrine and Collagen/ADP cartridges indicated no significant effect of PM_2.5_ and PM_10_ on platelet function. The effects of PM_2.5_ and PM_10_ on platelets and coagulation tests at the lag of one to 7 days are illustrated in Figure 3.

There were no reported cardiovascular incidents among the healthy individuals included in our study during the study period.

## 4 Discussion

The distinctive seasonal pollution phenomenon in Northern Thailand provides an opportunity to investigate the short-term effects of PM on adverse health outcomes. Despite efforts to protect themselves using face masks and/or indoor air purifiers, individuals unavoidably experience prolonged exposure to high concentrations of fine PM during periods of poor air quality. Past evidence has confirmed the link between high PM seasons and increased incidences of cardiovascular events, including myocardial infarction and stroke, in this region (49, 50). Several studies have reported that PM exposure enhances interleukin-6 (IL-6) and tumor necrotic factor-α (TNF-α) activation, contributing to the inflammatory process and resulting in thromboembolism (41). This study provides evidence regarding the short-term effects of ambient air pollution on hemostatic changes, which are the intermediary markers of cardiovascular risk in healthy individuals during seasonal variations in air pollution.

We observed a significant association between increased concentrations of PM_2.5_ and PM_10_ and the shortening of PT. This finding aligns with the results of other clinical studies (18, 58, 59), supporting the hypothesis that elevated concentrations of ambient PM may contribute to enhanced hypercoagulability. However, despite the statistical shortening of PT values, they remain within the normal range. Limited evidence links the PM-mediated PT shortening with clinically relevant thrombosis (18). Further studies should be conducted to investigate the clinical impact of pollution on thromboembolism.

A previous study reported a positive association between vWF antigen and the concentration of PM_2.5_, suggesting PM-induced vascular endothelial injury (60), while another study showed no significant correlation (61). Although, we observed a negative trend in vWF antigen, it was not statistically significant. Despite no significant change in vWF antigen levels due to PM, we interestingly found an increased function of vWF, as indicated by the ristocetin cofactor assay, with a strong association with short-term exposure to high ambient PM_2.5_ and PM_10_. Considering that vWF serves as the carrier of Factor VIII and protects against Factor VIII elimination in plasma (62), the increased vWF function resulting from elevated PM concentration in this study is consistent with the findings in a previous report in C57BL/6 mice, which showed a correlation between elevated Factor VIII levels and high PM_10_ concentrations (5). Further studies focusing on the effects of PM on vascular injury and coagulation factor assays could potentially elucidate the link between PM and vWF alterations.

We observed evidence of increased soluble P-selectin, reflecting heightened platelet activation resulting from high PM_2.5_ and PM_10_. These findings align with previous reports in healthy individuals (60, 63). Another study in diabetic patients also observed increased levels of soluble P-selectin and reported non-statistically significant increases in ADP-induced aggregation and decreases in collagen-induced aggregation and thromboxane B2 production with certain PM components (64). It has been reported that the closure time of the platelet function analyzer-100 (PFA-100) was shortened in hamsters after exposure to diesel exhaust particles (3, 65). In this study, we used PFA-200 to assess the effect of PM on platelet function and found no association with ambient PM levels. PM enhances platelet activation without inducing changes in the platelet function parameters measured by the PFA-200. This discrepancy might be because the PFA-200 is more effective in assessing the impact of anti-platelet agents rather than platelet activation and may not fully capture the platelet activation process. Another possible explanation could be the influence of PM dosage or the extent of platelet activation. Another hypothesis is that this may be due to a selective effect of PM on endothelial cells rather than directly on platelets. Moreover, platelet functions are intricate and governed by multiple mechanisms, ensuring that certain pathological functions are controlled by alternative mechanisms to maintain normal physiological function. Platelet aggregation tests or rotational thromboelastometry (ROTEM) may provide different results and more detailed insights into the PM-mediated effects. Future research should include detailed platelet function tests, such as platelet aggregation tests, ROTEM, and assessments of adhesion/expression of P-selectin or integrin for a more thorough understanding.

The mechanistic links between these PM-mediated hemostatic changes, including endothelial injury, endothelial function, platelet granule release and aggregation, coagulation factor levels, and fibrinolytic system should be explored. Significant clinical impacts need further clarification. Future research to prevent the health impact of PM and to develop novel prevention strategies is crucial. Emphasizing the adverse effects of inhaled ambient air pollution and addressing the global concern regarding increased PM levels is essential. Global policies are needed to mitigate PM production and improve global health.

The strength of this study lies in the unique seasonal variation of ambient air pollution in the area, which aligns with the study design that repeatedly evaluates hemostatic values in individuals over alterations in ambient pollution. However, there are several limitations. First, although we assumed that each volunteer was exposed to the same PM concentrations recorded at the Central Air Quality Monitoring Station, the actual dose of PM inhaled by each participant was not measured. Measuring the actual exposure of each individual would provide a more accurate interpretation of the PM-mediated effects and should be incorporated in the future studies.

Second, although we tried to control for confounding factors influencing the hemostatic parameters, such as age, body weight, height, and blood groups, our study did not control for individual factors such as diet and smoking status, as participants were encouraged to maintain their daily routines. Further research should incorporate these factors.

Third, the number of subjects was small, and some were unable to complete the four follow-up visits due to the COVID-19 pandemic.

Fourth, all participants were of Thai ethnicity. The impact of ambient particles may have varying outcomes in other ethnic groups, as the Asian population has lower incidences of thromboembolism than the Western population (66–68). Genetic differences across ethnic groups may influence susceptibility to PM exposure. Certain populations may have genetic predispositions affecting their response to environmental pollutants, altering the risk profile for cardiovascular events. Additionally, only healthy male participants were included this study. Ambient air pollution may have different hemostatic impacts on women due to hormonal differences affecting cardiovascular and hemostatic functions. Studies have shown that women might be more susceptible to air pollution-related cardiovascular events (69, 70).

Furthermore, the impact of ambient PM on hemostatic outcomes may differ, especially in populations with pre-existing conditions. The older adults, patients with cardiovascular diseases, cancer, and diabetes may have amplified or mitigated responses to PM exposure. The presence of chronic inflammation or compromised cardiovascular function could exacerbate the hemostatic changes induced by PM.

Finally, we did not assess inflammatory biomarkers such as IL-6, TNF- α, coagulation factor assays, and the fibrinolytic system, which may explain the mechanistic links between a hypercoagulable state and inhaled pollution. Future research should incorporate detailed inflammatory biomarkers and clinical outcomes of cardiovascular complications such as myocardial infarction and stroke to assess the comprehensive impact of PM exposure on individuals.

## 5 Conclusion

The results of this report indicate that short-term exposure to ambient PM_2.5_ and PM_10_ may potentially induce a prothrombotic state by enhancing platelet activation and von Willebrand function. Further studies investigating the adverse clinical outcomes of ambient PM on thrombotic complications should be conducted. Understanding the pathological mechanisms of how PM is associated with a hypercoagulable state would help provide possible resolutions to protect against these adverse outcomes. Global policies are required to limit the production of hazardous particles or reduce the impact of harmful ambient particles.

## Data availability statement

The raw data supporting the conclusions of this article will be made available by the authors, without undue reservation.

## Ethics statement

The studies involving humans were approved by Research Ethics Committee Faculty of Medicine, Chiang Mai University. The studies were conducted in accordance with the local legislation and institutional requirements. The participants provided their written informed consent to participate in this study.

## Author contributions

SH: Formal analysis, Writing – review & editing, Conceptualization, Methodology, Visualization, Writing – original draft. MS: Data curation, Software, Writing – review & editing. BS: Resources, Writing – review & editing. NL: Resources, Writing – review & editing. PN: Data curation, Project administration, Visualization, Writing – review & editing. SK: Data curation, Resources, Writing – review & editing. SW: Data curation, Project administration, Writing – review & editing. NP: Investigation, Validation, Writing – review & editing. PT: Investigation, Validation, Writing – review & editing. OW: Investigation, Writing – review & editing. RN: Investigation, Resources, Writing – review & editing. PK: Investigation, Resources, Writing – review & editing. PhP: Formal analysis, Methodology, Writing – review & editing. TP: Writing – review & editing. NH: Formal analysis, Supervision, Writing – review & editing. PoP: Writing – review & editing. TR: Writing – review & editing. CC-A: Supervision, Writing – review & editing. ER: Supervision, Writing – review & editing. AT: Supervision, Writing – review & editing. LN: Supervision, Writing – review & editing. SS: Investigation, Resources, Supervision, Writing – review & editing.

## References

[1] Lelieveld J Evans JS Fnais M Giannadaki D Pozzer A. The contribution of outdoor air pollution sources to premature mortality on a global scale. Nature. (2015) 525:367–71. doi: 10.1038/nature15371, PMID: 26381985

[2] Brook RD Newby DE Rajagopalan S. The global threat of outdoor ambient air pollution to cardiovascular health: time for intervention. JAMA Cardiol. (2017) 2:353–4. doi: 10.1001/jamacardio.2017.0032, PMID: 28241232

[3] Nemmar A Hoet PH Dinsdale D Vermylen J Hoylaerts MF Nemery B. Diesel exhaust particles in lung acutely enhance experimental peripheral thrombosis. Circulation. (2003) 107:1202–8. doi: 10.1161/01.cir.0000053568.13058.67, PMID: 12615802

[4] Nemmar A Hoylaerts MF Hoet PHM Vermylen J Nemery B. Size effect of intratracheally instilled particles on pulmonary inflammation and vascular thrombosis. Toxicol Appl Pharmacol. (2003) 186:38–45. doi: 10.1016/s0041-008x(02)00024-8, PMID: 12583991

[5] Mutlu GM Green D Bellmeyer A Baker CM Burgess Z Rajamannan N. Ambient particulate matter accelerates coagulation via an IL-6-dependent pathway. J Clin Invest. (2007) 117:2952–61. doi: 10.1172/jci30639, PMID: 17885684 PMC1978421

[6] Riva DR Magalhães CB Lopes AA Lanças T Mauad T Malm O. Low dose of fine particulate matter (PM2.5) can induce acute oxidative stress, inflammation and pulmonary impairment in healthy mice. Inhalation Toxicology. (2011) 257–267. doi: 10.3109/08958378.2011.566290 PMID: 21506876

[7] Sawyer K Mundandhara S Ghio AJ Madden MC. The effects of ambient particulate matter on human alveolar macrophage oxidative and inflammatory responses. J Toxicol Environ Health A. (2010) 73:41–57. doi: 10.1080/15287390903248901 19953419

[8] Danielsen PH Møller P Jensen KA Sharma AK Wallin H Bossi R. Oxidative stress, DNA damage, and inflammation induced by ambient air and wood smoke particulate matter in human A549 and THP-1 cell lines. Chem Res Toxicol. (2011) 24:168–84. doi: 10.1021/tx100407m, PMID: 21235221

[9] SALVI S BLOMBERG A RUDELL B KELLY F SANDSTRÖM T HOLGATE S T. Acute inflammatory responses in the airways and peripheral blood after short-term exposure to diesel exhaust in healthy human volunteers. Am J Respir Crit Care Med. (1999) 159:702–9. doi: 10.1164/ajrccm.159.3.9709083, PMID: 10051240

[10] Hantrakool S Kumfu S Chattipakorn SC Chattipakorn N. Effects of particulate matter on inflammation and thrombosis: past evidence for future prevention. Int J Environ Res Public Health. (2022) 19:771. doi: 10.3390/ijerph19148771, PMID: 35886623 PMC9317970

[11] Budinger GR McKell JL Urich D Foiles N Weiss I Chiarella SE. Particulate matter-induced lung inflammation increases systemic levels of PAI-1 and activates coagulation through distinct mechanisms. PLoS One. (2011) 6:e18525. doi: 10.1371/journal.pone.0018525, PMID: 21494547 PMC3073968

[12] Khosravipour M Safari-Faramani R Rajati F Omidi F. The long-term effect of exposure to respirable particulate matter on the incidence of myocardial infarction: a systematic review and meta-analysis study. Environ Sci Pollut Res Int. (2022) 29:42347–71. doi: 10.1007/s11356-022-18986-6, PMID: 35355187

[13] Zhu W Cai J Hu Y Zhang H Han X Zheng H. Long-term exposure to fine particulate matter relates with incident myocardial infarction (MI) risks and post-MI mortality: a meta-analysis. Chemosphere. (2021) 267:128903. doi: 10.1016/j.chemosphere.2020.128903 33213879

[14] Xu R Huang S Shi C Wang R Liu T Li Y. Extreme temperature events, fine particulate matter, and myocardial infarction mortality. Circulation. (2023) 148:312–23. doi: 10.1161/circulationaha.122.063504 37486993

[15] de Bont J Jaganathan S Dahlquist M Persson Å Stafoggia M Ljungman P. Ambient air pollution and cardiovascular diseases: an umbrella review of systematic reviews and meta-analyses. J Intern Med. (2022) 291:779–800. doi: 10.1111/joim.13467, PMID: 35138681 PMC9310863

[16] Li XY Yu XB Liang WW Yu N Wang L Ye XJ. Meta-analysis of association between particulate matter and stroke attack. CNS Neurosci Ther. (2012) 18:501–8. doi: 10.1111/j.1755-5949.2012.00325.x, PMID: 22672304 PMC6493517

[17] Huang K Liang F Yang X Liu F Li J Xiao Q. Long term exposure to ambient fine particulate matter and incidence of stroke: prospective cohort study from the China-PAR project. BMJ. (2019) 367:l6720. doi: 10.1136/bmj.l6720, PMID: 31888885 PMC7190010

[18] Baccarelli A Martinelli I Zanobetti A Grillo P Hou LF Bertazzi PA. Exposure to particulate air pollution and risk of deep vein thrombosis. Arch Intern Med. (2008) 168:920–7. doi: 10.1001/archinte.168.9.920, PMID: 18474755 PMC3093962

[19] Montiel-Davalos A Gonzalez-Villava A Rodriguez-Lara V Montano LF Fortoul TI Lopez-Marure R. Vanadium pentoxide induces activation and death of endothelial cells. J Appl Toxicol. (2012) 32:26–33. doi: 10.1002/jat.1695, PMID: 21721017

[20] Bumroongkit C Liwsrisakun C Deesomchok A Pothirat C Theerakittikul T Limsukon A. Correlation of air pollution and prevalence of acute pulmonary embolism in northern Thailand. Int J Environ Res Public Health. (2022) 19:2808. doi: 10.3390/ijerph191912808, PMID: 36232104 PMC9566050

[21] Hamra GB Guha N Cohen A Laden F Raaschou-Nielsen O Samet JM. Outdoor particulate matter exposure and lung cancer: a systematic review and meta-analysis. Environ Health Perspect. (2014) 122:906–11. doi: 10.1289/ehp/1408092, PMID: 24911630 PMC4154221

[22] Li J Li WX Bai C Song Y. Particulate matter-induced epigenetic changes and lung cancer. Clin Respir J. (2017) 11:539–46. doi: 10.1111/crj.12389, PMID: 26403658 PMC7310573

[23] Pritchett N Spangler EC Gray GM Livinski AA Sampson JN Dawsey SM. Exposure to outdoor particulate matter air pollution and risk of gastrointestinal cancers in adults: a systematic review and Meta-analysis of epidemiologic evidence. Environ Health Perspect. (2022) 130:36001. doi: 10.1289/ehp9620, PMID: 35234536 PMC8890324

[24] Zare Sakhvidi MJ Lequy E Goldberg M Jacquemin B. Air pollution exposure and bladder, kidney and urinary tract cancer risk: a systematic review. Environ Pollut. (2020) 267:115328. doi: 10.1016/j.envpol.2020.115328 32871482

[25] Pengchai P Chantara S Sopajaree K Wangkarn S Tengcharoenkul U Rayanakorn M. Seasonal variation, risk assessment and source estimation of PM 10 and PM10-bound PAHs in the ambient air of Chiang Mai and Lamphun, Thailand. Environ Monit Assess. (2009) 154:197–218. doi: 10.1007/s10661-008-0389-0, PMID: 18688736

[26] Othman M Latif MT Hamid HHA Uning R Khumsaeng T Phairuang W. Spatial-temporal variability and heath impact of particulate matter during a 2019-2020 biomass burning event in Southeast Asia. Sci Rep. (2022) 12:7630. doi: 10.1038/s41598-022-11409-z, PMID: 35538095 PMC9086666

[27] Kliengchuay W Cooper Meeyai A Worakhunpiset S Tantrakarnapa K. Relationships between meteorological parameters and particulate matter in Mae Hong Son Province, Thailand. Int J Environ Res Public Health. (2018) 15:2801. doi: 10.3390/ijerph15122801, PMID: 30544675 PMC6313660

[28] Vinitketkumnuen U Kalayanamitra K Chewonarin T Kamens R. Particulate matter, PM 10 & PM 2.5 levels, and airborne mutagenicity in Chiang Mai, Thailand. Mutat Res. (2002) 519:121–31. doi: 10.1016/s1383-5718(02)00130-4 12160897

[29] Ho RC Zhang MW Ho CS Pan F Lu Y Sharma VK. Impact of 2013 south Asian haze crisis: study of physical and psychological symptoms and perceived dangerousness of pollution level. BMC Psychiatry. (2014) 14:81. doi: 10.1186/1471-244x-14-81, PMID: 24642046 PMC3995317

[30] Duc HN Bang HQ Quan NH Quang NX. Impact of biomass burnings in Southeast Asia on air quality and pollutant transport during the end of the 2019 dry season. Environ Monit Assess. (2021) 193:565. doi: 10.1007/s10661-021-09259-9, PMID: 34383149

[31] Yin S Wang X Zhang X Guo M Miura M Xiao Y. Influence of biomass burning on local air pollution in mainland Southeast Asia from 2001 to 2016. Environ Pollut. (2019) 254:112949. doi: 10.1016/j.envpol.2019.07.117, PMID: 31376599

[32] Pothirat C Chaiwong W Liwsrisakun C Bumroongkit C Deesomchok A Theerakittikul T. Influence of particulate matter during seasonal smog on quality of life and Lung function in patients with chronic obstructive pulmonary disease. Int J Environ Res Public Health. (2019) 16:106. doi: 10.3390/ijerph16010106, PMID: 30609775 PMC6339110

[33] Varapongpisan T Frank TD Ingsrisawang L. Association between out-patient visits and air pollution in Chiang Mai, Thailand: lessons from a unique situation involving a large data set showing high seasonal levels of air pollution. PLoS One. (2022) 17:e0272995. doi: 10.1371/journal.pone.0272995, PMID: 35980887 PMC9387779

[34] Supasri T Gheewala SH Macatangay R Chakpor A Sedpho S. Association between ambient air particulate matter and human health impacts in northern Thailand. Sci Rep. (2023) 13:12753. doi: 10.1038/s41598-023-39930-9, PMID: 37550356 PMC10406826

[35] Surit P Wongtanasarasin W Boonnag C Wittayachamnankul B. Association between air quality index and effects on emergency department visits for acute respiratory and cardiovascular diseases. PLoS One. (2023) 18:e0294107. doi: 10.1371/journal.pone.0294107, PMID: 37972204 PMC10653395

[36] Pothirat C Chaiwong W Liwsrisakun C Bumroongkit C Deesomchok A Theerakittikul T. Acute effects of air pollutants on daily mortality and hospitalizations due to cardiovascular and respiratory diseases. J Thorac Dis. (2019) 11:3070–83. doi: 10.21037/jtd.2019.07.37, PMID: 31463136 PMC6687987

[37] Pothirat C Chaiwong W Liwsrisakun C Bumroongkit C Deesomchok A Theerakittikul T. The short-term associations of particular matters on non-accidental mortality and causes of death in Chiang Mai, Thailand: a time series analysis study between 2016-2018. Int J Environ Health Res. (2021) 31:538–47. doi: 10.1080/09603123.2019.1673883, PMID: 31569960

[38] Nakharutai N Traisathit P Thongsak N Supasri T Srikummoon P Thumronglaohapun S. Impact of residential concentration of PM2.5 analyzed as time-varying covariate on the survival rate of Lung Cancer patients: a 15-year hospital-based study in upper northern Thailand. Int J Environ Res Public Health. (2022) 19:4521. doi: 10.3390/ijerph19084521, PMID: 35457386 PMC9026284

[39] Wichmann HE. Diesel exhaust particles. Inhal Toxicol. (2007) 19:241–4. doi: 10.1080/08958370701498075 17886072

[40] Combes A Franchineau G. Fine particle environmental pollution and cardiovascular diseases. Metabolism. (2019) 100:153944. doi: 10.1016/j.metabol.2019.07.008 31610849

[41] Signorelli SS Oliveri Conti G Zanobetti A Baccarelli A Fiore M Ferrante M. Effect of particulate matter-bound metals exposure on prothrombotic biomarkers: a systematic review. Environ Res. (2019) 177:108573. doi: 10.1016/j.envres.2019.108573, PMID: 31323394

[42] Bevan GH Al-Kindi SG Brook R Rajagopalan S. Ambient air pollution and atherosclerosis: recent updates. Curr Atheroscler Rep. (2021) 23:63. doi: 10.1007/s11883-021-00958-9, PMID: 34417890 PMC8379601

[43] Liang S Zhang J Ning R Du Z Liu J Batibawa JW. The critical role of endothelial function in fine particulate matter-induced atherosclerosis. Part Fibre Toxicol. (2020) 17:61. doi: 10.1186/s12989-020-00391-x, PMID: 33276797 PMC7716453

[44] Bai Y Sun Q. Fine particulate matter air pollution and atherosclerosis: mechanistic insights. Biochim Biophys Acta. (2016) 1860:2863–8. doi: 10.1016/j.bbagen.2016.04.030, PMID: 27156486

[45] Hayes RB Lim C Zhang Y Cromar K Shao Y Reynolds HR. PM2.5 air pollution and cause-specific cardiovascular disease mortality. Int J Epidemiol. (2020) 49:25–35. doi: 10.1093/ije/dyz114, PMID: 31289812 PMC7124502

[46] Wu J Tian Y Wu Y Wang Z Wu Y Wu T. Seasonal association between ambient fine particulate matter and venous thromboembolism in Beijing, China: a time-series study. Environ Sci Pollut Res Int. (2021) 28:32795–801. doi: 10.1007/s11356-021-13035-0 33634399

[47] Martinelli N Girelli D Cigolini D Sandri M Ricci G Rocca G. Access rate to the emergency department for venous thromboembolism in relationship with coarse and fine particulate matter air pollution. PLoS One. (2012) 7:e34831. doi: 10.1371/journal.pone.0034831, PMID: 22509360 PMC3324538

[48] Franchini M Mengoli C Cruciani M Bonfanti C Mannucci PM. Association between particulate air pollution and venous thromboembolism: a systematic literature review. Eur J Intern Med. (2016) 27:10–3. doi: 10.1016/j.ejim.2015.11.012, PMID: 26639051

[49] Mueller W Loh M Vardoulakis S Johnston HJ Steinle S Precha N. Ambient particulate matter and biomass burning: an ecological time series study of respiratory and cardiovascular hospital visits in northern Thailand. Environ Health. (2020) 19:77. doi: 10.1186/s12940-020-00629-3, PMID: 32620124 PMC7333306

[50] Jarernwong K Gheewala SH Sampattagul S. Health impact related to ambient particulate matter exposure as a spatial health risk map case study in Chiang Mai, Thailand. Atmos. (2023) 14:261. doi: 10.3390/atmos14020261

[51] Emmerechts J de Vooght V Haenen S Loyen S van kerckhoven S Hemmeryckx B. Thrombogenic changes in young and old mice upon subchronic exposure to air pollution in an urban roadside tunnel. Thromb Haemost. (2012) 108:756–68. doi: 10.1160/TH12-03-0161 22872007

[52] Wu Z Liu MC Liang M Fu J. Sirt1 protects against thrombomodulin down-regulation and lung coagulation after particulate matter exposure. Blood. (2012) 119:2422–9. doi: 10.1182/blood-2011-04-350413, PMID: 22262770

[53] Liang S Zhao T Hu H Shi Y Xu Q Miller MR. Repeat dose exposure of PM(2.5) triggers the disseminated intravascular coagulation (DIC) in SD rats. Sci Total Environ. (2019) 663:245–53. doi: 10.1016/j.scitotenv.2019.01.346, PMID: 30711591 PMC6398278

[54] Nemmar A Nemery B Hoet PH Vermylen J Hoylaerts MF. Pulmonary inflammation and thrombogenicity caused by diesel particles in hamsters: role of histamine. Am J Respir Crit Care Med. (2003) 168:1366–72. doi: 10.1164/rccm.200306-801OC, PMID: 12969870

[55] Tabor CM Shaw CA Robertson S Miller MR Duffin R Donaldson K. Platelet activation independent of pulmonary inflammation contributes to diesel exhaust particulate-induced promotion of arterial thrombosis. Part Fibre Toxicol. (2015) 13:6. doi: 10.1186/s12989-016-0116-x, PMID: 26857113 PMC4746929

[56] Nemmar A Subramaniyan D Yasin J Ali BH. Impact of experimental type 1 diabetes mellitus on systemic and coagulation vulnerability in mice acutely exposed to diesel exhaust particles. Part Fibre Toxicol. (2013) 10:14. doi: 10.1186/1743-8977-10-14 23587270 PMC3641025

[57] Chiarella SE Soberanes S Urich D Morales-Nebreda L Nigdelioglu R Green D. β₂-adrenergic agonists augment air pollution-induced IL-6 release and thrombosis. J Clin Invest. (2014) 124:2935–46. doi: 10.1172/jci75157, PMID: 24865431 PMC4071386

[58] Tang L Shi S Wang B Liu L Yang Y Sun X. Effect of urban air pollution on CRP and coagulation: a study on inpatients with acute exacerbation of chronic obstructive pulmonary disease. BMC Pulm Med. (2021) 21:296. doi: 10.1186/s12890-021-01650-z, PMID: 34537026 PMC8449878

[59] Bonzini M Tripodi A Artoni A Tarantini L Marinelli B Bertazzi PA. Effects of inhalable particulate matter on blood coagulation. J Thromb Haemost. (2010) 8:662–8. doi: 10.1111/j.1538-7836.2009.03694.x, PMID: 19922434 PMC3093960

[60] Rich DQ Kipen HM Huang W Wang G Wang Y Zhu P. Association between changes in air pollution levels during the Beijing Olympics and biomarkers of inflammation and thrombosis in healthy young adults. JAMA. (2012) 307:2068–78. doi: 10.1001/jama.2012.3488, PMID: 22665106 PMC4049319

[61] Croft DP Cameron SJ Morrell CN Lowenstein CJ Ling F Zareba W. Associations between ambient wood smoke and other particulate pollutants and biomarkers of systemic inflammation, coagulation and thrombosis in cardiac patients. Environ Res. (2017) 154:352–61. doi: 10.1016/j.envres.2017.01.027, PMID: 28167447 PMC5375102

[62] Federici AB. The factor VIII/von Willebrand factor complex: basic and clinical issues. Haematologica. (2003) 88:Erep02. PMID: 12826528

[63] Xu H Wang T Liu S Brook RD Feng B Zhao Q. Extreme levels of air pollution associated with changes in biomarkers of atherosclerotic plaque vulnerability and Thrombogenicity in healthy adults. Circ Res. (2019) 124:e30–43. doi: 10.1161/CIRCRESAHA.118.313948, PMID: 30661461

[64] Becerra AZ Georas S Brenna JT Hopke PK Kane C Chalupa D. Increases in ambient particulate matter air pollution, acute changes in platelet function, and effect modification by aspirin and omega-3 fatty acids: a panel study. J Toxicol Environ Health A. (2016) 79:287–98. doi: 10.1080/15287394.2016.1157539, PMID: 27029326 PMC4919023

[65] Nemmar A Hoylaerts MF Hoet PH Nemery B. Possible mechanisms of the cardiovascular effects of inhaled particles: systemic translocation and prothrombotic effects. Toxicol Lett. (2004) 149:243–53. doi: 10.1016/j.toxlet.2003.12.061, PMID: 15093270

[66] Zeng Y Si H Wu Y Yang J Zhou Z Kang P. The incidence of symptomatic in-hospital VTEs in Asian patients undergoing joint arthroplasty was low: a prospective, multicenter, 17,660-patient-enrolled cohort study. Knee Surg Sports Traumatol Arthrosc. (2019) 27:1075–82. doi: 10.1007/s00167-018-5253-3 30386998

[67] Jang MJ Bang SM Oh D. Incidence of venous thromboembolism in Korea: from the Health Insurance Review and Assessment Service database. J Thromb Haemost. (2011) 9:85–91. doi: 10.1111/j.1538-7836.2010.04108.x, PMID: 20942850

[68] Lazo-Langner A Liu K Shariff S Garg AX Ray JG. Immigration, region of origin, and the epidemiology of venous thromboembolism: a population-based study. Res Pract Thromb Haemost. (2018) 2:469–80. doi: 10.1002/rth2.12113, PMID: 30046751 PMC6046583

[69] Qu Y Pan Y Niu H He Y Li M Li L. Short-term effects of fine particulate matter on non-accidental and circulatory diseases mortality: a time series study among the elder in Changchun. PLoS One. (2018) 13:e0209793. doi: 10.1371/journal.pone.0209793 30596713 PMC6312390

[70] Bell ML Zanobetti A Dominici F. Evidence on vulnerability and susceptibility to health risks associated with short-term exposure to particulate matter: a systematic review and meta-analysis. Am J Epidemiol. (2013) 178:865–76. doi: 10.1093/aje/kwt090, PMID: 23887042 PMC3775545

