# Supplementary material for: The effects of ambient particulate matter air pollution on platelets and hemostasis
Source: Front Public Health. 2024 Jul 18;12:1410406. doi: 10.3389/fpubh.2024.1410406 (PMC11292950; doi:10.3389/fpubh.2024.1410406)
Supplement: Supplementary file 1 [file Data_Sheet_1.docx]

Supplementary Material

# Supplementary Tables

**Supplementary Table 1.** Distribution of Mean Pollutant Concentrations and Other Meteorological Parameters for Each Participant on Lag Days One to Seven in Each Study Visit.

| Meteorological parameters | Lag Days | Week 0 (N=30) | | | Week 4^th^ (N=30) | | | Week 8^th^ (N=28) | | | Week 12^th^ (N=26) | | | Overall (N=114) | | |
| --- | --- | --- | --- | --- | --- | --- | --- | --- | --- | --- | --- | --- | --- | --- | --- | --- |
|  |  | Median | IQR1 | IQR3 | Median | IQR1 | IQR3 | Median | IQR1 | IQR3 | Median | IQR1 | IQR3 | Median | IQR1 | IQR3 |
| PM2.5, µg/m^3^ | 1 | 68.04 | 52.96 | 83.25 | 35.25 | 13.88 | 54.23 | 14.36 | 6.75 | 22.88 | 6.21 | 5.63 | 7.69 | 18.63 | 8.42 | 54.23 |
|  | 2 | 71.65 | 49.50 | 85.48 | 43.00 | 15.09 | 51.34 | 13.63 | 8.03 | 20.99 | 6.13 | 5.70 | 7.84 | 18.13 | 8.37 | 51.34 |
|  | 3 | 80.52 | 52.30 | 98.09 | 46.49 | 15.31 | 55.24 | 13.43 | 8.03 | 21.20 | 6.36 | 6.03 | 7.56 | 21.20 | 8.13 | 54.14 |
|  | 4 | 80.22 | 52.92 | 107.71 | 47.85 | 15.08 | 55.07 | 15.48 | 8.36 | 20.99 | 6.64 | 2.65 | 7.25 | 20.99 | 8.28 | 52.92 |
|  | 5 | 89.16 | 50.72 | 109.46 | 47.87 | 16.80 | 55.76 | 17.52 | 8.59 | 19.97 | 6.43 | 5.73 | 7.33 | 19.97 | 7.87 | 52.57 |
|  | 6 | 91.18 | 51.37 | 105.17 | 46.74 | 18.26 | 54.54 | 17.64 | 8.46 | 19.27 | 6.28 | 5.81 | 7.46 | 19.27 | 7.57 | 51.37 |
|  | 7 | 92.09 | 52.39 | 103.12 | 47.07 | 19.18 | 52.68 | 17.09 | 8.63 | 19.18 | 6.24 | 6.01 | 7.11 | 21.35 | 7.31 | 52.39 |
| PM10, µg/m^3^ | 1 | 98.09 | 80.23 | 100.83 | 52.13 | 25.63 | 77.83 | 25.92 | 15.05 | 36.86 | 15.96 | 13.83 | 18.33 | 31.38 | 18.21 | 80.23 |
|  | 2 | 91.84 | 72.43 | 110.46 | 64.89 | 28.36 | 76.79 | 25.59 | 16.29 | 34.29 | 16.22 | 14.09 | 17.93 | 30.96 | 18.34 | 76.79 |
|  | 3 | 98.94 | 78.96 | 121.79 | 68.04 | 28.28 | 78.96 | 26.01 | 17.74 | 34.32 | 16.17 | 15.12 | 17.71 | 34.32 | 17.78 | 79.37 |
|  | 4 | 100.42 | 79.50 | 124.82 | 69.76 | 27.79 | 79.84 | 28.03 | 18.15 | 34.29 | 15.74 | 14.79 | 17.27 | 34.29 | 17.99 | 79.84 |
|  | 5 | 110.42 | 80.12 | 136.33 | 69.42 | 29.91 | 79.46 | 30.50 | 18.26 | 32.97 | 15.80 | 14.43 | 17.19 | 32.97 | 17.44 | 80.12 |
|  | 6 | 111.78 | 81.88 | 137.73 | 68.42 | 30.77 | 80.15 | 30.46 | 18.06 | 32.17 | 15.61 | 14.63 | 16.82 | 32.17 | 16.93 | 81.88 |
|  | 7 | 117.04 | 80.55 | 132.02 | 68.59 | 32.05 | 80.55 | 29.88 | 18.49 | 32.05 | 15.48 | 14.85 | 16.28 | 33.80 | 16.82 | 83.08 |
| CO, ppm | 1 | 1.01 | 0.91 | 1.06 | 0.73 | 0.56 | 0.83 | 0.46 | 0.43 | 0.56 | 0.42 | 0.37 | 0.46 | 0.58 | 0.43 | 0.91 |
| NO_2_, ppb | 1 | 11.67 | 0.00 | 20.33 | 0.00 | 0.00 | 0.00 | 0.00 | 0.00 | 0.00 | 0.00 | 0.00 | 0.00 | 0.00 | 0.00 | 0.93 |
| Temperature, °C | 1 | 29.79 | 28.76 | 31.29 | 30.59 | 28.19 | 31.40 | 30.27 | 27.92 | 31.40 | 28.99 | 28.10 | 30.46 | 29.91 | 28.24 | 31.06 |
| Humidity, % | 1 | 45.05 | 42.88 | 47.38 | 54.00 | 47.00 | 57.75 | 60.00 | 55.38 | 71.25 | 72.75 | 67.25 | 78.63 | 55.75 | 47.38 | 70.00 |
| Wind Velocity, km/hr | 1 | 20.37 | 16.67 | 24.08 | 20.37 | 18.52 | 25.93 | 25.00 | 20.37 | 28.71 | 22.00 | 19.00 | 27.70 | 22.22 | 18.52 | 25.93 |

**Supplementary Table 2.** Mean Blood Cell Count and Hemostatic Parameters at Each Time Point.

| **Blood and Hemostatic Parameters** | **Mean Blood cell counts and Hemostatic Values in Each Time Point**  **Compared to baseline (mean ±SD, p value)** | | | |
| --- | --- | --- | --- | --- |
|  | **Week 0 (N=30)** | **Week 4 (N=30)** | **Week 8 (N=28)** | **Week 12 (N=26)** |
| Hb, g/dL | 14.70 ± 0.84 | 14.73 ± 0.96  0.8395 | 14.59 ± 1.00  0.7713 | 14.47 ± 0.83  0.1594 |
| Hct, % | 43.84 ± 2.09 | 43.85± 2.46  0.9775 | 43.41 ± 2.89  0.4608 | 43.10 ± 1.99  0.0162 |
| WBC, cells/cu.mm. | 6,091 ± 1,626 | 3,084 ± 1,624  0.9746 | 5,811 ± 1,281  0.2536 | 5,824 ± 1,283  0.1335 |
| Platelet, cells/cu.mm. | 276,766 ± 68,281 | 281,600 ± 59,962  0.3846 | 275,785 ± 53,305  0.3754 | 283,461 ± 60,222  0.2132 |
| PT, seconds | 10.73 ± 0.56 | 11.07 ± 1.08  0.1051 | 11.48 ± 1.88  0.0540 | 11.16 ± 0.79  0.0077 |
| aPTT, seconds | 32.61 ± 2.30 | 33.42 ± 2.94  0.1139 | 33.58 ± 3.66  0.1711 | 31.99 ± 2.97  0.1014 |
| vWF antigen, % | 122.20 ± 40.50 | 122.35 ± 44.89  0.9714 | 122.96 ± 43.08  0.9193 | 132.60 ± 43.24  0.0845 |
| vWF Ristocetin cofactor, % | 89.25 ± 29.05 | 83.86 ± 31.49  0.1487 | 84.53 ± 30.11  0.1259 | 82.99 ± 25.27  0.0152 |
| PFA200: Epinephrine/Collagen, seconds | 117.86 ± 26.91 | 112.48 ± 24.90  0.3394 | 115.93 ± 26.54  0.7648 | 114.00 ± 33.28  0.6480 |
| PFA200: ADP/Collagen, seconds | 90.47  41.70 | 80.37 ± 12.92  0.2099 | 84.68 ± 15.51  0.4872 | 82.54 ± 12.41  0.3336 |
| Soluble P-selectin, ng/ml | 91.06 ± 35.39 | 82.66 ± 32.33  0.2043 | 85.28 ± 39.66  0.3522 | 80.55 ± 45.10  0.3441 |

**
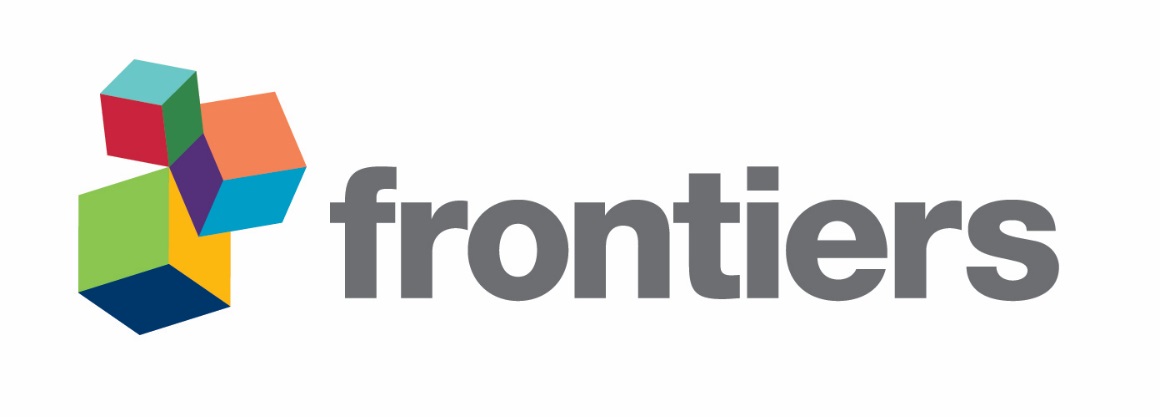
**
